# Systematic Review of Current Approaches to Tibia Plateau: Best Clinical Evidence

**DOI:** 10.7759/cureus.27183

**Published:** 2022-07-23

**Authors:** Gur Aziz Singh Sidhu, Jamie Hind, Neil Ashwood, Harjot Kaur, Hannah Bridgwater, Shyam Rajagopalan

**Affiliations:** 1 Trauma and Orthopaedics, University Hospitals of Derby and Burton NHS Foundation Trust, Burton, GBR; 2 Trauma and Orthopaedics, Walsall Manor Hospital, Burton, GBR; 3 Anesthesia, Queens Elizabeth Hospital, London, GBR; 4 Junior Anatomy Demonstrator, Human Anatomy Centre, Department of Physiology, Development and Neuroscience, University of Cambridge, Cambridge, GBR

**Keywords:** orif, proximal tibia fracture, knee approach, anterolateral approach, tibia plateau fracture

## Abstract

If not treated adequately, tibia plateau fractures result in premature osteoarthritis and lifelong disability. The advent of newer implants and techniques to improve outcomes has necessitated the development of different surgical approaches. A Medline and EMBASE search (June 2020) was conducted to identify publications during the last 10 years that focused on surgical approaches for proximal tibia fractures/ tibia plateau management. A total of 2107123 and 2715399 articles were found related to fractures in this area with 133 and 103 review articles looking at the approach on MEDLINE and EMBASE, respectively. This article reviews the continued development of the surgical approaches to aid understanding for surgeons and identify areas for future research to help improve outcomes. Although the anterolateral approach is the most commonly applied surgical technique, having the knowledge of newer approaches (medial, posteromedial, posterolateral, or direct posterior) in the armamentarium is necessary to treat the vast array of fracture patterns. There has been a shift amongst trauma surgeons of using a combination of approaches for complex tibia plateau fractures.

## Introduction and background

Tibia plateau fractures are complex knee injuries that can be difficult to stabilize and require a thorough knowledge of the anatomy to facilitate reconstruction, early mobilization, and prevent long-term complications [1-3]. Historically, open reduction and plate fixation was achieved through a single midline incision with extensive exposure of the fracture, increased soft tissue damage, and disruption to the remaining osseous blood supply resulting in a compromised surgical outcome [4]. However, preservation of the blood supply can be achieved through the use of external fixators and Ilizarov frames, where safe zones of wire placement need to be recognized [5]. Some authors have found the issues of pin site care and non-union to be higher in this group [5]. The advent of locking plates, the use of raft screws, and minimal access techniques have reduced overall morbidity [6,7].

The treatment of such fractures has been traditionally based on Schatzker’s classification of the injury [8]. The use of computer tomography (CT) scan has allowed a more detailed recognition of the injury and planning through a more considered approach by understanding the anatomy involved more clearly [9]. The goals of treatment remain restoration of articular congruity, and limb alignment with stable fixation to allow early range of motion of the knee [8]. This article aims to review the anatomical principles underpinning the surgical approaches providing aid in understanding and fixing these complex fractures.

## Review

Proximal tibia/plateau fractures are common and difficult-to-manage injuries. Numerous approaches have been mentioned in the literature for the treatment of such fractures. The question for this study was what is the best approach to be followed for tibia plateau fixation after complications like increased infection rate as well as inadequate fixation encountered in the local audit. 

A MEDLINE and EMBASE search ( 01/06/2020 to 18/06/2020) was conducted to identify publications during the last 10 years that focused on surgical approaches for proximal tibia fractures management [10]. After an exhaustive search on MEDLINE, the following results were obtained 1957 articles related to tibial plateau fracture, 2582 articles related to proximal tibial plateau fractures, and 4243 articles with either of two, 1958630 articles related to surgical fixation of tibial plateau, and 133 review articles related to tibial fractures. Similarly, EMBASE search yielded 583 articles with tibial plateau fracture, 1191 articles with proximal tibia fractures, 2817 articles with either of the two, 84172 articles related to fracture fixation, 47976 related to knee surgery, 2668172 related to surgical fixation of the tibia, 2715399 with either of the above and 103 review articles. 

**Figure 1:**
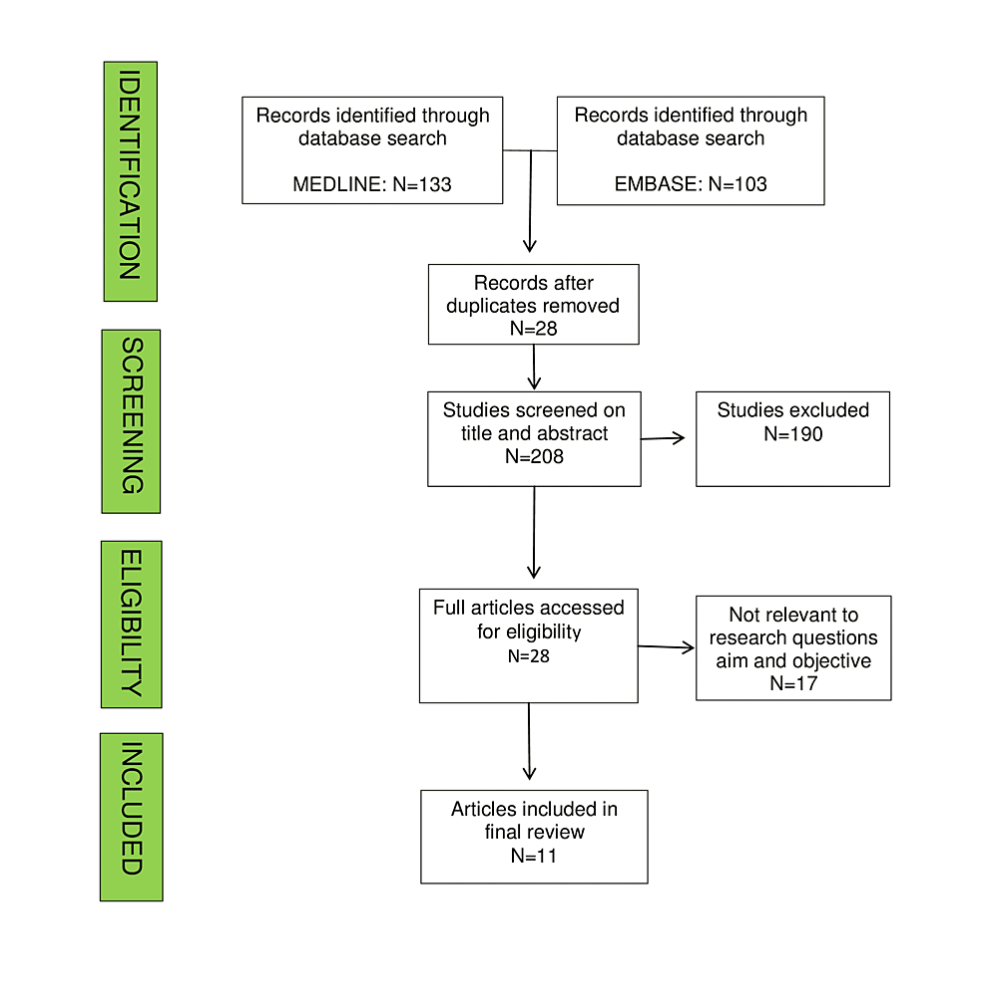
Schematic protocol for our study

We included studies that meet the following criteria: review articles, English language, tibia plateau fracture approaches, approaches around knee or proximal tibia, and studies on surgical techniques for proximal tibia fixation. Studies were excluded if inclusion criteria were not met or patients with tibia shaft fractures, polytrauma, basic science or animal studies, or editorial/opinion/case report with less than ten patients (Figure 1).

Two authors (GS and JH) independently screened all retrieved items by title and abstract, followed by full text using the predetermined selection criteria. Disagreements were resolved through discussion with the authors according to the Greenhalgh and Peacock method [11]. The review process highlighted the most commonly used approaches over the medial and lateral aspects of the knee. These were performed on embalmed body donors by an anatomist. The approaches were then dissected further to reveal related anatomy. These dissections were photographed for the anatomical relations of each approach.

Data on study characteristics and design, classification, surgical technique, revision surgeries, and treatment outcomes were extracted by a single author (GS) from studies in a spreadsheet. Statistical analysis was performed using the Statistical Package for Social Sciences (SPSS). The primary outcomes evaluated in this review were different surgical approaches available till date for fixation of proximal tibia plateau fractures. Analysis was achieved through an iterative process using a narrative synthesis [12]. 

Lateral approaches

Anterolateral Approach

**Figure 2:**
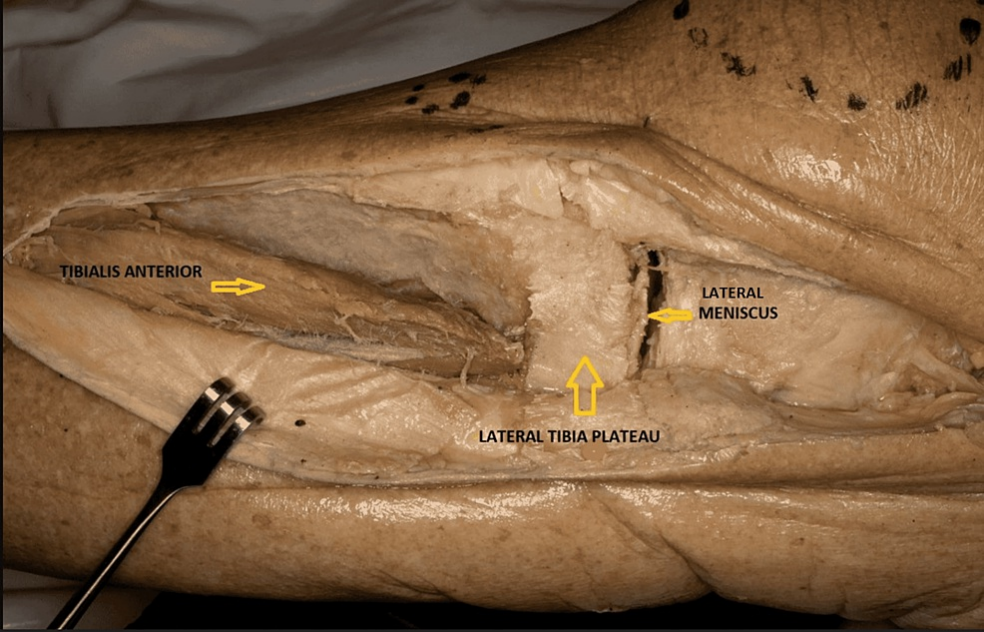
Anterolateral approach for proximal tibia plateau (Cadaveric donor dissection)

It is the most popular approach followed by a majority of trauma surgeons for fixing tibia plateau fractures [13,14]. This approach is typically used for Schatzker's type III, V, and VI fracture patterns [13,14]. A lazy “S” incision is made starting from the iliotibial (IT) band, curving around the Gerdy's tubercle (GT), and distally over the tibial crest (few centimeters laterally). Proximally, the IT band is cut in line with its fibers and the anterior compartment fascia is incised distal to the Gerdy’s tubercle, towards the tibial crest. An interval is developed between the IT band and the joint capsule. Distally, the tibialis anterior muscle is retracted laterally to expose the tibia [13] (Figure 2). The potent advantages include simple technique, little surgical damage, easy body position during the operation, and easy reproduction of the approach to remove implants later [14]. An extended anterolateral approach is more useful for fixing certain posterolateral tibial plateau fractures than a direct posterior approach requiring careful preoperative planning [15]. 

Posterolateral Approach

It is used for coronal fractures with displaced posterolateral fragments or fractures not addressed through the anterolateral approach [16,17,18]. The original technique was described by Lobenhoffer and co-workers and different variations have appeared relating to fibular osteotomy [16-22]. This approach can be performed in a prone, supine, or lateral position [17-22]. Proximally, a 4-inch longitudinal incision starts over the medial aspect of the biceps femoris (BF) tendon and distally to the posteromedial border of the fibula. Skin, subcutaneous tissue, and popliteal fascia are dissected. The common peroneal nerve (CPN) is identified as it lies medial to the BF tendon and needs to be dissected to avoid CPN palsy. The plane of dissection is between the lateral gastrocnemius (LG) and the BF. Distally, the soleus can be bluntly dissected to provide exposure to the proximal tibia and posterolateral ligaments. Moreover, popliteus and the lateral horn of the arcuate ligament can be incised to improve the exposure. The potent advantage of fibular osteotomy is good exposure to the joint line; however, it increases patient morbidity [18].

Medial approaches

**Figure 3:**
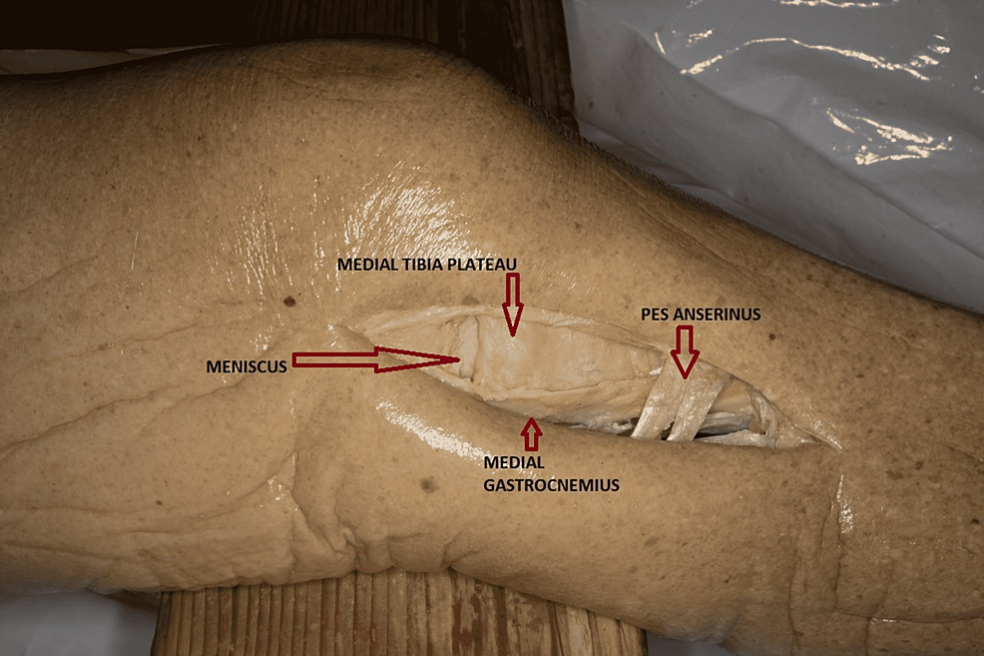
Medial approach for proximal tibia plateau (Cadaveric donor dissection)

This approach is usually used for Schatzker type IV fractures. However, recently, with the increase in the complexity of fractures and introduction of the dual plating technique, this approach is used for Schatzker type V fractures [16,23,24]. The incision starts from the medial femoral epicondyle proximally and extends over the pes anserinus. With the knee flexed at 15 degrees, a straight incision 10 cm in length is made to expose 3 layered structures [25]. The neurovascular structures (saphenous nerve and vein) lie posterior to the incision. After incising the subcutaneous tissues, the sartorius fascia and pes anserinus tendons lie in the first layer. Further down is the superficial medial collateral ligament (MCL) in the second layer, and the deep MCL forms the third layer (Figure 3) [26].

Posteromedial Approach

This is an ideal approach for shear fractures of the medial tibia plateau, such as Moore type I fracture or AO 41 C-type fractures [23,24]. The patient can be positioned supine or prone [23,27-31]. Better ergonomics (allows axial traction) and gravity (helps in reduction) are the potential benefits of prone positioning [23,31].

Posteromedial Approach (Supine)

In this approach, the leg is externally rotated before a longitudinal incision beginning 3 cm proximal to the joint line and is then extended along the posterior margin of the tibia [28-30]. It is essential to protect the saphenous nerve and great saphenous vein during the incision. In line with the incision, sartorius fascia is incised. The pes anserinus tendons are retracted anteriorly and the medial gastrocnemius (MG) and soleus are retracted posteriorly exposing the junction between semimembranosus (SM) and MCL. The perisoteum is incised longitudinally over the posterior border of the MCL. The popliteus muscle is elevated subperiostally off the posterior tibia allowing visualization of fracture.

Posteromedial Approach (Prone)

**Figure 4:**
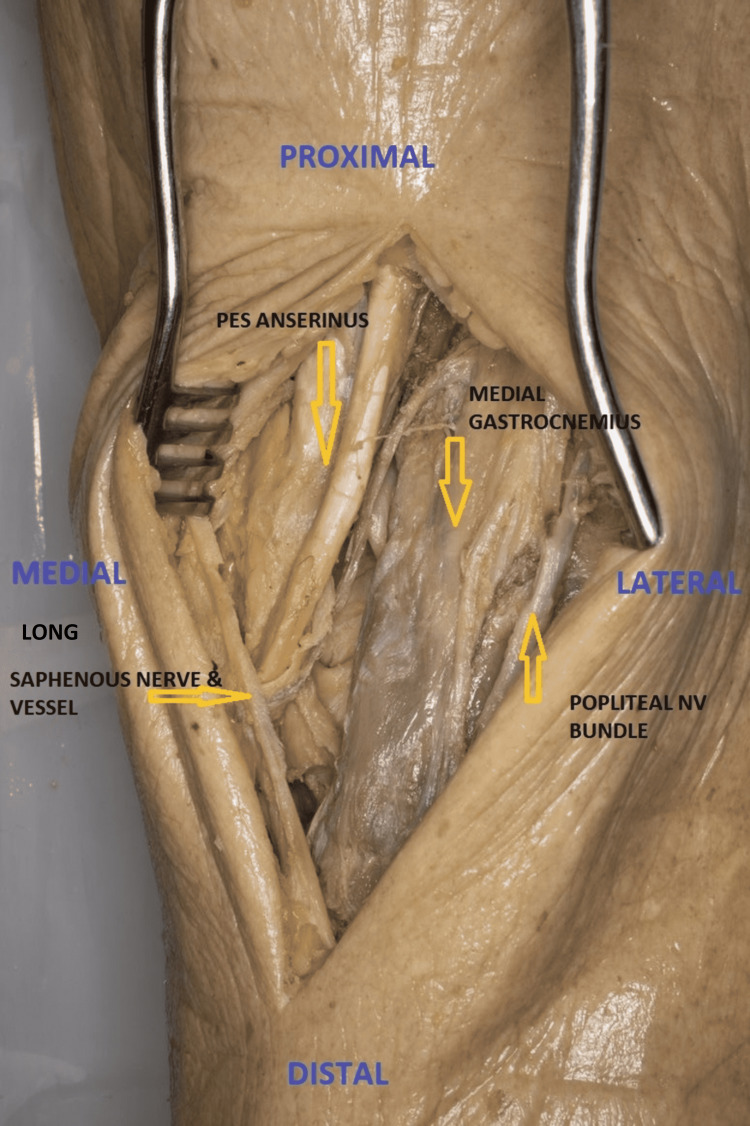
Posteromedial approach to proximal tibia [Prone](Cadaveric donor dissection)

A posteromedial approach is mostly used in Moore type I tibial plateau fractures as an alternative to the posterior approach [23,31]. The patient is positioned prone with a sandbag under the contralateral hip. In prone position, the incision is posterior and lateral as compared to the supine position approach. Incision (8 cm) extends longitudinally above the joint line and extends along the medial border of the medial gastrocnemius (MG). The MG with the neurovascular bundle is retracted laterally to develop an interval between the medial gatrocnemius and semimembranosus. Pes anserinus dissection is avoided in this approach. The subperiosteal dissection of the popliteal fascia and popliteus expose the bone [29,31] (Figure 4).

Direct Posterior Approach

**Figure 5:**
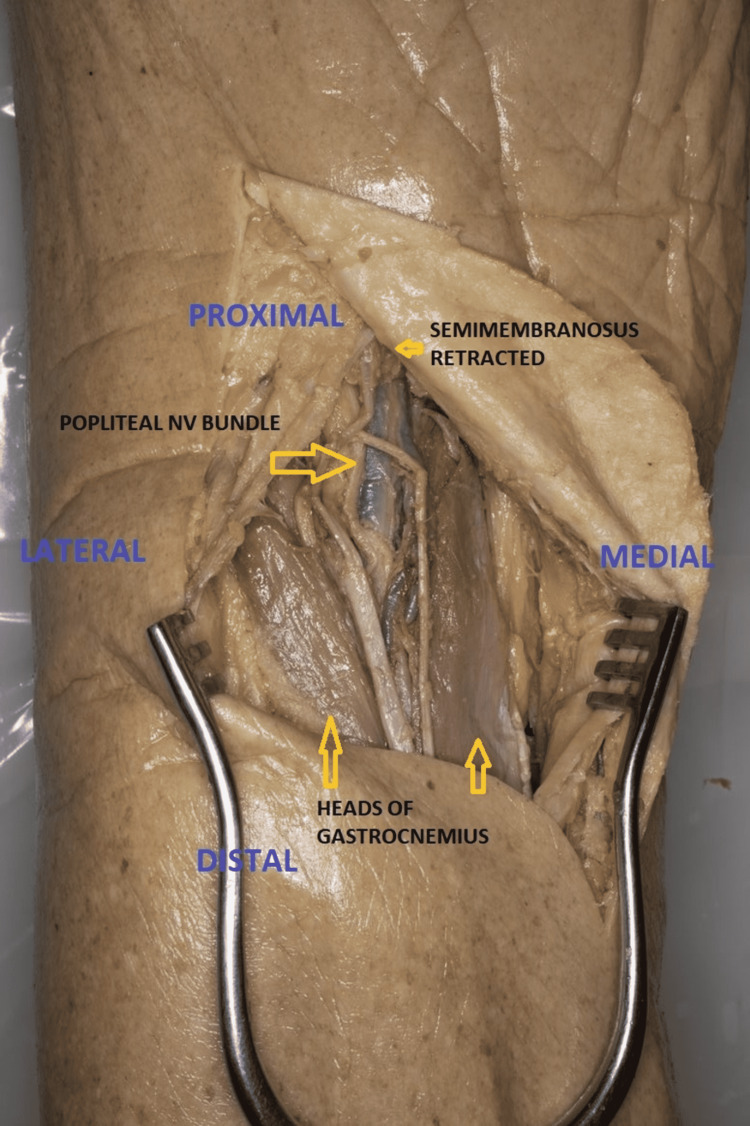
Direct posterior approach to proximal tibia [prone](Cadaveric donor dissection)

The use of direct posterior approach is limited to shear fractures of the posterior plateau or avulsion fractures of the posterior cruciate ligament (PCL) [26,27,32]. The three-column fixation concept uses this approach to fix the posterior column fracture element of any injury [32]. An “S” shaped incision is started over BF proximally curving over the popliteal fossa and extending distally along the medial head of the gastrocnemius. Neurovascular structures (lesser saphenous vein and sural nerve) can be identified near the joint line and keeping the incision close to the medial head of the gastrocnemius protects the sural nerve. The tibial nerve is followed up proximally up to the junction of SM and BF. The popliteal artery lies medial to the nerve but the popliteal vein lies lateral to the artery proximally and crosses medial to the artery distally. The CPN (lying on the medial side of BF) is dissected and retracted laterally (Figure 5). The deep dissection is similar to the posteromedial approach. The popliteal neurovascular structures are at risk of injury during this approach [33].

The treatment of bicondylar tibial plateau fracture is a serious challenge in terms of soft-tissue complications, fracture morphology, early mobilization, and maintenance of reduction postoperatively. Some of these fractures may need dual plating through an anterolateral and/or medial/posteromedial/posterior approach depending upon the fracture morphology [33-36]. A newer angiosome sparing approach or anterior perforator sparing (APS) approach has been developed along the anterior and superior margin of the anterior tibial angiosome (ATA) [36].

## Conclusions

The proximal tibia can be approached from anterolateral, medial, or posterior aspects depending upon the fracture morphology. An ideal surgical approach should allow visualization and reduction of the fracture with minimal risk of complications. So, a combined approach (anterolateral with posteromedial/medial or posterolateral with medial) is an ideal approach that leads to a good functional outcome of tibial plateau fracture fixation with minimal complications. Moreover, its the detailed analysis of the fracture pattern that should dictate the choice of the appropriate approach rather than the other way around. Trauma surgeons should have the knowledge of different approaches as these can be used alone or in combination to treat a vast array of fracture patterns that occur in the proximal tibia.

## References

[1] Manidakis N, Dosani A, Dimitriou R (2010). Tibial plateau fractures: functional outcome and incidence of osteoarthritis in 125 cases. Int Orthop.

[2] Rademakers MV, Kerkhoffs GM, Sierevelt IN, Raaymakers EL, Marti RK (2007). Operative treatment of 109 tibial plateau fractures: Five- to 27-year follow-up results. J Orthop Trauma.

[3] Kokkalis ZT, Iliopoulos ID, Pantazis C, Panagiotopoulos E (2016). What's new in the management of complex tibial plateau fractures?. Injury.

[4] Hannouche D, Duparc F, Beaufils P The arterial vascularization of the lateral tibial condyle: Anatomy and surgical applications. Surg Radiol Anat.

[5] Bove F, Sala F, Capitani P, Thabet AM, Scita V, Spagnolo R (2018). Treatment of fractures of the tibial plateau (Schatzker VI) with external fixators versus plate osteosynthesis. Injury.

[6] Biggi F, Di Fabio S, D’Antimo C, Trevisani S (2010). Tibial plateau fractures: Internal fixation with locking plates and the MIPO technique. Injury.

[7] Messina M, Herbert B, Mauffrey C (2013). The use of arthroscopy to assist reduction of depressed tibial plateau fractures. Curr Orthop Pract.

[8] Kfuri M, Schatzker J (2018). Revisiting the Schatzker classification of tibial plateau fractures. Injury.

[9] Castiglia MT, Nogueira-Barbosa MH, Messias AM, Salim R, Fogagnolo F, Schatzker J, Kfuri M (2018). The impact of computed tomography on decision making in tibial plateau fractures. J Knee Surg.

[10] Booth A (2016). Searching for qualitative research for inclusion in systematic reviews: a structured methodological review. Syst Rev.

[11] Greenhalgh T, Peacock R (2005). Effectiveness and efficiency of search methods in systematic reviews of complex evidence: audit of primary sources. BMJ.

[12] Aveyard Aveyard, H. H., Payne Payne, S. S., Preston Preston, N N A Post-graduate’s Guide to Doing a Literature Review: in Health and Social Care. Open University Press.

[13] Padanilam TG, Ebraheim NA, Frogameni A (1995). Meniscal detachment to approach lateral tibial plateau fractures. Clin Orthop Relat Res.

[14] Hake ME, Goulet JA (2016). Open reduction and internal fixation of the tibial plateau through the anterolateral approach. J Orthop Trauma.

[15] Chen HW and Luo CF (2015). Extended anterolateral approach for treatment of posterolateral tibial plateau fractures improves operative procedure and patient prognosis. Int J Clin Exp Med.

[16] Lobenhoffer P, Gerich T, Bertram T, Lattermann C, Pohlemann T, Tscheme H (1997). Treatment of posterior tibial plateau fractures via posteromedial and posterolateral exposures. Unfallchirurg.

[17] Tao J, Hang DH, Wang QG, Gao W, Zhu LB, Wu XF, Gao KD (2008). The posterolateral shearing tibial plateau fracture: treatment and results via a modified posterolateral approach. Knee.

[18] Solomon LB, Stevenson AW, Baird RP, Pohl AP (2010). Posterolateral transfibular approach to tibial plateau fractures: technique, results, and rationale. J Orthop Trauma.

[19] Carlson DA (2005). Posterior bicondylar tibial plateau fractures. J Orthop Trauma.

[20] Chang SM, Zheng HP, Li HF, Jia YW, Huang YG, Wang X, Yu GR (2009). Treatment of isolated posterior coronal fracture of the lateral tibial plateau through posterolateral approach for direct exposure and buttress plate fixation. Arch Orthop Trauma Surg.

[21] Frosch KH, Balcarek P, Walde T, Stürmer KM (2010). A new posterolateral approach without fibula osteotomy for the treatment of tibial plateau fractures. J Orthop Trauma.

[22] Kumar G, Peterson N, Narayan B (2011). Bicondylar tibial fractures: Internal or external fixation?. Indian J Orthop.

[23] Barei DP, O'Mara TJ, Taitsman LA, Dunbar RP, Nork SE (2008). Frequency and fracture morphology of the posteromedial fragment in bicondylar tibial plateau fracture patterns. J Orthop Trauma.

[24] Warren LA, Marshall JL, Girgis F (1974). The prime static stabilizer of the medical side of the knee. J Bone Joint Surg Am.

[25] Phisitkul P, James SL, Wolf BR, Amendola A (2006). MCL injuries of the knee: Current concepts review.. Iowa Orthop J.

[26] Burks RT, Schaffer JJ (1990). A simplified approach to the tibial attachment of the posterior cruciate ligament. Clin Orthop Relat Res.

[27] Georgiadis GM (1994). Combined anterior and posterior approaches for complex tibial plateau fractures. J Bone Joint Surg Br.

[28] Galla M, Lobenhoffer P (2003). [The direct, dorsal approach to the treatment of unstable tibial posteromedial fracture-dislocations]. Unfallchirurg.

[29] Weil YA, Gardner MJ, Boraiah S, Helfet DL, Lorich DG (2008). Posteromedial supine approach for reduction and fixation of medial and bicondylar tibial plateau fractures. J Orthop Trauma.

[30] Vasanad GH, Antin SM, Akkimaradi RC, Policepatil P, Naikawadi G (2013). Surgical management of tibial plateau fractures - A clinical study. J Clin Diagn Res.

[31] Hu SJ, Chang SM (2015). Comments on: Prone and direct posterior approach for management of posterior column tibial plateau fractures of K.-C. Lin, Y.-W. Tarng, G.-Y. Lin, S.-W. Yang, C.-J. Hsu, J.-H. Renn published in Orthop Traumatol Surg Res 2015;101(4):477-482. Orthop Traumatol Surg Res.

[32] Medvecky MJ, Noyes FR (2005). Surgical approaches to the posteromedial and posterolateral aspects of the knee. J Am Acad Orthop Surg.

[33] Bhalotia AP: Ingle MV, Koichade MR (2018). Necessity of dual plating in bicondylar tibial plateau fracture dislocations: A prospective case series. J Orthop Traumatol Rehab.

[34] Musahl V, Tarkin I, Kobbe P, Tzioupis C, Siska PA, Pape HC (2009). New trends and techniques in open reduction and internal fixation of fractures of the tibial plateau. J Bone Joint Surg Br.

[35] Zhang Y, Fan DG, Ma BA, Sun SG (2012). Treatment of complicated tibial plateau fractures with dual plating via a 2-incision technique. Orthopedics.

[36] Solomon LB, Boopalan PR, Chakrabarty A, Callary SA (2014). Can tibial plateau fractures be reduced and stabilised through an angiosome-sparing antero-lateral approach?. Injury.

